# Clustering Users to Determine the Most Suitable Gamification Elements

**DOI:** 10.3390/s22010308

**Published:** 2021-12-31

**Authors:** Alejandro Blanco-M, Ruth S. Contreras-Espinosa, Jordi Solé-Casals

**Affiliations:** Data and Signal Processing Group, University of Vic-Central University of Catalonia, c/de la Laura 13, 08500 Vic, Catalonia, Spain; alejandro.blanco@uvic.cat

**Keywords:** gamification, user profile, game elements, game genre, clustering

## Abstract

The use of gamification elements has extended from being a complement for a product to being integrated into multiple public services to motivate the user. The first drawback for service designers is choosing which gamification elements are appropriate for the intended audience, in addition to the possible incompatibilities between gamification elements. This work proposes a clustering technique that enables mapping different user profiles in relation to their preferred gamification elements. Additionally, by mapping the best cluster for each gamification element, it is possible to determine the preferred game genre. The article answered the following research questions: What is the relationship between the genre of the game and the element of gamification? Different user groups (profiles) for each gamification element? Results indicate that there are cases where the users are divided between those who agree or disagree. However, other elements present a great heterogeneity in the number of groups and the levels of agreement.

## 1. Introduction

The use of gamification elements has gradually extended from being a mere complement to a given product or brand, to becoming an incentive in public services to motivate users to interact more frequently and generate more engagement.

Gamification as a strategy has been proven partially successful in domains such as the business context, where different gamification features can be integrated into products, websites, or services [1]. Gamification implies bringing about similar positive experiences to those of games and, consequently, affects user behavior and cognitive processes [2]. Mainly inspired by games, gamification commonly applies game mechanics. Authors such as [3,4] make a distinction between three categories of gamification mechanics and game design which are directly related to gaming motivation: (1) the immersion-related, (2) the achievement-related, and (3) the social-related dimensions. Immersion-related features pursue to immerse the user in a self-directed activity and include storytelling, avatars, or role-play as game mechanics. On the other hand, achievement-related features seek to increase the user’s sense of accomplishment and include challenges, badges, missions, leaderboards, goals, or progression metrics as game mechanics. Finally, social-related features pursue to enable user social interaction and include collaboration and cooperation structures as game mechanics.

According to [5,6], to understand the principles of gamification it is necessary to comprehend the sources of human motivation. As stated by [7], user motivation can be divided into two distinct categories according to its origin: intrinsic and extrinsic motivation. Recent studies analyze these two concepts in connection with the current issue of gamification [8,9,10]. Zichermann and Cunningham [9] indicate that intrinsic motivation is achieved through activities that generate challenges or are enjoyable. On the other hand, extrinsic motivation is only achieved through rewards, either of a material nature, such as gifts, access to exclusive areas or discounts, or virtual items, like exclusive badges that lead to a distinctive status within and across communities, or that contribute to earning special recognition in different social networks.

In addition, there are different game/gamification frameworks that may help designers select the appropriate game elements, such as:

- **Mechanics–Dynamics–Aesthetics (MDA)** by [11] is a model composed of three elements: (1) game mechanics are the basic actions that players can undertake in a game, responses, algorithms, stored data, etc.; (2) game dynamics are the run-time behavior of the previously defined mechanics in response to the player input and to the interaction among other types of mechanics; lastly, (3) game aesthetics are the emotional responses produced in the player.
- **Six D’s (Six Steps to Gamification)** by [12] is a model based on six points: (1) define the objectives that you want to achieve; (2) delineate the target behaviors that you expect from the users; (3) describe your players’ profile (interest, what drives them); (4) devise activity loops (the process that the users have to follow); (5) do not forget the fun (think what makes your users return); and (6) deploy the appropriate tools (how the interaction will be measured, score systems, badge assignations, etc.).
- **GAME** by [13] is a framework with four components: (1) gather what information will be collected; (2) design the best solution for your goals and the experience of your users based on the information that you have; (3) monitor user activity and goals, iterate improvements; and (4) enrich your solution over time to match the changes in society. This methodology evolved into the RAMPS motivation model and, later, into the User Types Hexad Scale, which is used to identify the types of users.
- **Octalysis** by [14] is a framework that focuses on human design rather than functional design. This framework is depicted in an octagon shape determined by the core drivers of motivation. According to the author, the right side of the octagon reflects intrinsic motivation factors, and the left side reflects the extrinsic motivation.
- **Gamification Model Canvas** by [15]: this model is based on the Business Model Canvas and the MDA mentioned previously, and it is another relevant, flexible, and agile tool that enables representing in a single page all the necessary elements, tasks, and expected results of the gamified environment.

The inclusion of gamification elements is gaining importance because technologies such as Augmented Reality (AR) technology, for example, have become an essential element in today’s digital life, changing the way users interact with different services. AR enables the superposition of virtual 3D objects in a real 3D environment thanks to devices equipped with a camera and a screen through which the citizen lives the immersive experience offered by the AR. AR aims to enrich the real environment with virtual information, creating the opportunity to offer real-time and collaborative input and it is increasingly common in public services and gamification elements can become an excellent addition to AR for tourism applications that require the user’s on-site visit, such as tours, shopping, and mapping different places of the city [16].

The decisions that designers take upon using some of these frameworks are normally based on the description and suppositions about the public of the service and their own expectations. Therefore, the first drawback encountered by the game designer is to match their expectations with the gamification elements which are appropriate for the target audience. In addition, the designer may be unaware of the existing interdependencies and synergies among different gamification elements. Through a questionnaire designed to determine the profile of the users of interest, we collected data to answer the following research questions:

- What is the relationship between the genre of the game and the element of gamification?

We wanted to examine whether users who prefer a particular type of game genre respond more positively to certain types of gamification elements. For example, we could expect that someone who typically plays adventure games will positively respond to missions and story elements.

- Different user groups (profiles) for each gamification element?

We wanted to determine if for each type of gamification element, a similar number of user profiles are generated, or if there is a greater heterogeneity of profiles for some gamification elements than others. Additionally, what type of elements have a greater number of profiles? Are the profiles balanced, or is there a group that has a notable majority?

Authors will first review existing research on gamification elements. Parts of this data have been used in previous publications [17], with more forthcoming. The following table (Table 1) provides an overview of the game elements of interest addressed in this work.

## 2. Materials and Methods

### 2.1. Data Collection and Processing

**Figure 1:**
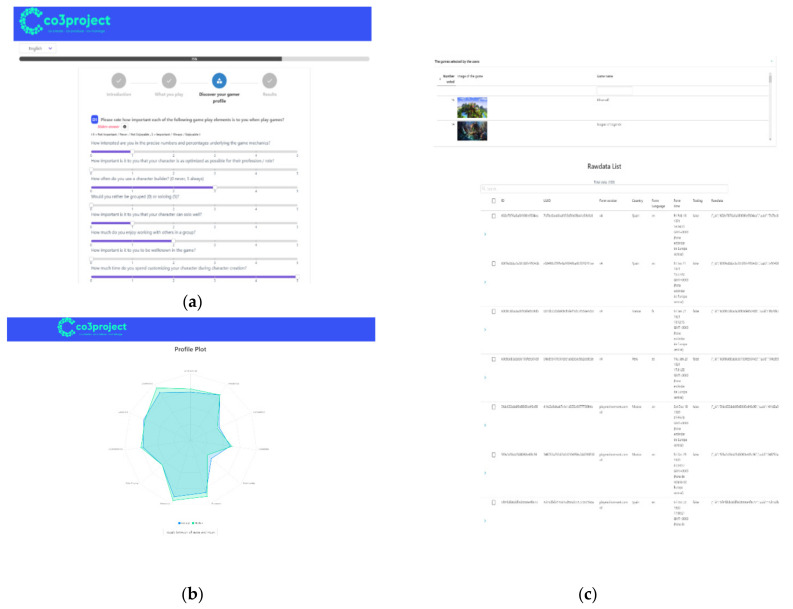
Screenshots of the web interface created for data collection. (**a**) shows a portion of the questions with the sliding selector, from 1 to 5. (**b**) shows the user’s profile in relation to the gamification elements. (**c**) shows the source data stored in a database.

Data collection was carried out through an online form at https://www.projectco3.eu/wp-content/gamificationformv4/ (accessed on 10 December 2021) that contained questions about the location, age, preferred games and the gamification questions to capture the participant’s profile (citizens). A screenshot of the questionnaire interface is shown in Figure 1. The gamification questions have been adapted from the paper by [16] having a base material to work with. The original questions of Nick Yee use the weights defined into a table to assign the player profile category [18]. In this case, we have not used those weights, and we carried out the analysis using the points from each question individually and grouped them by correlation to the key questions selected by us. We have processed the data with R code inside the Rstudio (R) [19] tool. This tool allows us to carry out clustering models using K-means modules and generate interactive graphs and reports using the GGPLOT library.

After carrying out the data collection, we noticed that some users had not answered some important questions that were crucial for the intended, and thus were excluded from the sample. obtaining N = 138 complete answers to the more than 46 questions. Of these 46 questions, 39 are of the Likert scale type; that is, the answer ranges from a value of 1 to 5, with 1 being less in agreement with the question and 5 more in agreement.

### 2.2. Determine the Key Questions

To carry out the analysis, we must associate the gamification elements with one key question, which is highly related to the concept. This step is critical since there is a human factor, and it is based on the interpretation from each of the question and the definition of each gamification element.

Then, after determining the key question, we proceed with applying a correlation algorithm to find out other questions related to responses. Next, a correlation matrix is made, and those questions that get a value bigger than 0.5 (medium correlation) are selected.

### 2.3. Determining the Groups of Users

Once we have the list of key questions–elements, we can determine if people respond similarly to each gamification element. For this reason, we apply an unsupervised data analysis technique of the clustering type known as K-Means [20].

The K-Means technique is a grouping technique in a certain number of groups (K). The elements in each group share similar characteristics (they respond similarly to the questions) and are, at the same time, separated from other groups that do not share characteristics. The K-Means algorithm determines whether they are similar or different by using a distance measure (Euclidean distance in our case). The algorithm has an iterative operation repeated as many times as there are elements to be classified/moved from the group. The technique needs to know in advance the number of groups that they want to generate (K).

**Figure 2:**
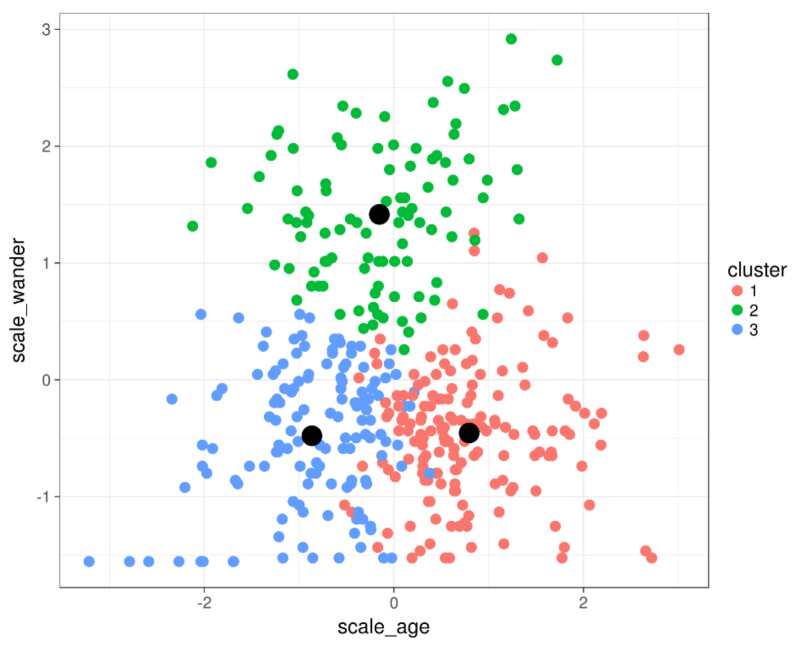
Result from clustering algorithm for three clusters indicated in three colors (red, green, blue). The centroids for each cluster are indicated with a black dot.

The K-Means algorithm repeats the following steps until there is no changes in terms of the dot cluster (color). Figure 2 can help to understand the following explanation:

1. The first time, the K central points (centroids), indicated at the Figure 2 in black, are set in a random position.
2. For each dot (individual) the Euclidian distance is calculated to each centroid of each cluster, determined in step 1, then the dot cluster (color) is set in concordance to the nearest centroid (smallest Euclidian distance).
3. Recalculate the centroids based on the dots (individuals) that are clustered to each centroid. The calculation is performed by taking the average position between all the members of the current centroid-cluster
4. Repeat from step 2.

**Figure 3:**
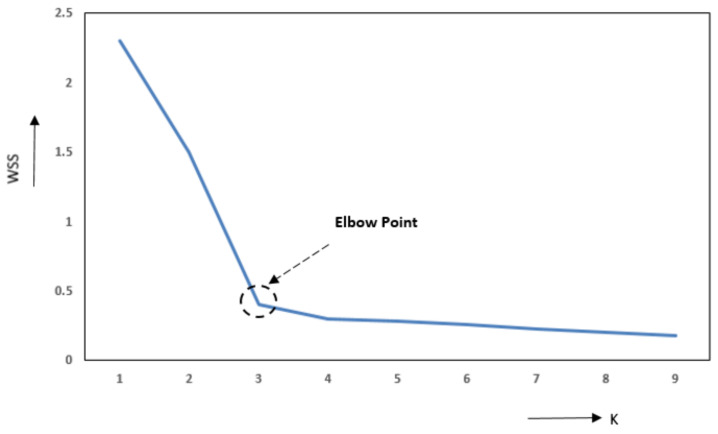
The evolution of the WSS vs. the number of clusters (K), indicating with an arrow the optimal point of change (Elbow Point) in the value of K = 3.

It is important to determine well in advance the number of groups/clusters (K) for each gamification element to obtain the maximum detail about our data. To determine K, we used the technique known as “Elbow” [21]. The “Elbow” method is based on representing the total variation of the clusters internally (total intracluster variation (or sum of the total square within the cluster (WSS)) [22] versus the number of clusters. Figure 3 depicts the evolution of the WSS vs. the number of clusters for the data in Figure 2. The lower the WSS, the better the quality of each cluster, the greater the number of clusters, the less grouped profiles we will have, which differs from the objective. That is why it is important to obtain a compromise point between the WSS and the cluster number, and that is why the “Elbow” allows us to determine the optimal point.

**Figure 4:**
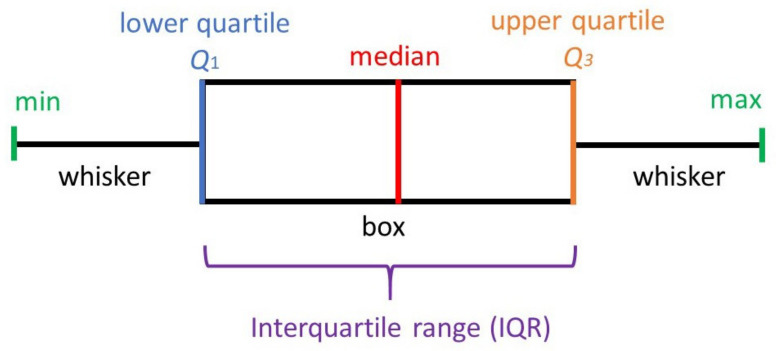
A taxonomy of a box plot. The figure shows six indicators: min, max, median, Q1, Q2, Q3, and the IQR in a reduced figure.

To visualize the clustering results, we use a graphical tool such as the Boxplots [23]. Boxplots condense statistical information in a small space, showing the three quartiles, the distance between the first and last quartiles, the median, and the extreme values. Figure 4 shows the physical taxonomy of the box plot.

**Figure 5:**
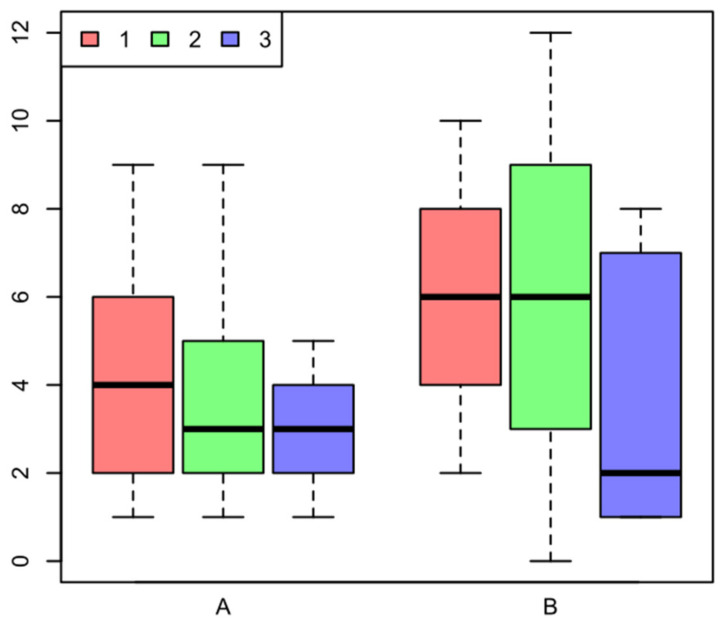
Example of clustering population comparison by using boxplots in terms of two variables (A and B).

The simplicity of the box plot provides a quick way to visually compare the results between clusters (groups). Taking the Figure 5 as an example with topics A/B and three clusters (1 red, 2 green, 3 blue), we are able to detect differences by simply comparing those. For instance, in the same figure for the topic A, we can observe that the blue boxplot is shorter than the others, which indicates that the individuals inside that cluster have more or less the same opinion (punctuation) about the topic A, also the same blue boxplot, indicates that the max value is less than six, which indicates that this group is less extreme in terms of the opinion. In comparison, the red boxplot representing the red cluster, the answers are more heterogenic, and the range is bigger, indicating a diversity of opinions about the topic A.

### 2.4. Determining the Game Genre Related to Each Gamification Element

Picking the best cluster for each gamification element and the statistics of the game genre played by the user, we can determine the preferred gamification element for each player type. To start the analysis, we have to pick up the best cluster. The best cluster is determined individually for each gamification element. The cluster with the highest average (mean) value is the selected one. Those scores are the responses rates facilitated by the users from the form questions. The best cluster is composed in theory, with the users that most agrees with the questions related to the current gamification element.

Next, for each of those best clusters, we collected the statistics about the game genre that the players inside each cluster play. We were able to construct the relation between gamification element and game genre in the case of our population (dataset).

## 3. Results

We have divided this section in partial results, following the style of the previous sections with different subsections. Each of the steps have the explanation of the result found and how it has been used inside the study. The results are incremental, which means that the new partial result takes the results of the previous steps as required information.

### 3.1. Determine the Key Questions

Starting with selecting the “key question”, as described in step 2, we have built the table (Table 2) that relates each element to the key question. The table indicates at the first column the gamification element that is being in consideration. The second column, key question, shows the question that we have determined as the representative for that gamification element. This means that the title includes a definition of the gamification element. The third column indicates the related questions to the key question that is determined by correlation. This is calculated by evaluating the correlations of all the questions to the key question, and only those that exceeded the 50% are selected. You can check the correlation value for each related question at the fourth column.

To be able to understand the text inside column two and three, you must check the Table 3 which contains the relation between the short title, which is the term that we are using inside the study for referencing the question.

This section may be divided by subheadings. It should provide a concise and precise description of the experimental results, their interpretation, as well as the experimental conclusions that can be drawn.

**Figure 6:**
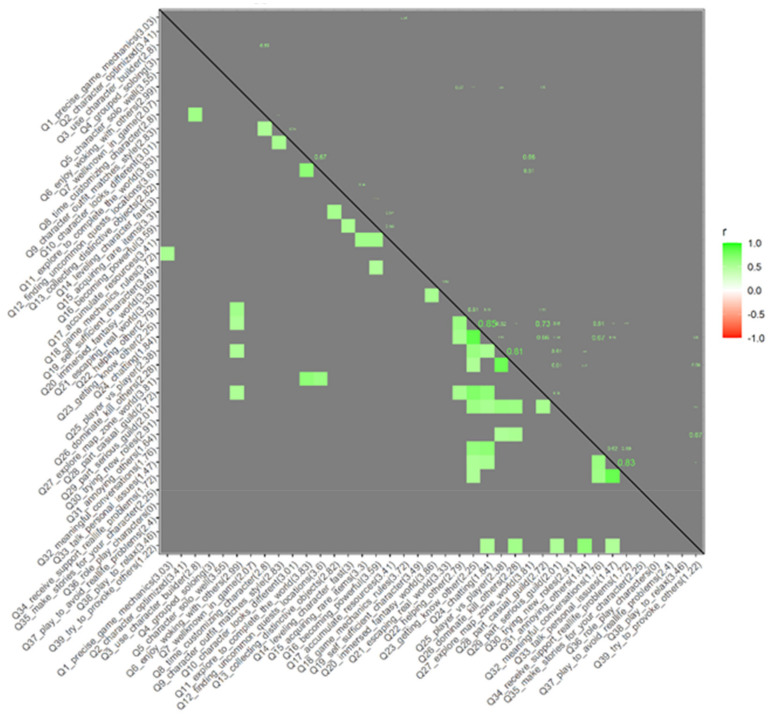
Correlations (r) greater than 0.5 in a matrix. Correlation indicated with colors under the diagonal and in numbers above the diagonal.

Figure 6 shows the correlation between all the questions that have more than 0.5 of correlation factor. All the questions are shown here, and most of the correlations are near the diagonal, which means the questions are related to the previous one.

### 3.2. Determining the Groups of Users

Continuing with the results on the clusters determined for each gamification element as indicated in point 3 of the methodology, we have found multiple amounts of groupings (clusters) of different sizes. Those differences in terms of sizes and number of clusters cannot be compared between gamification elements, because of the use of different questions for each gamification element. Next, we must analyze individually by observing the differences inside each category.

#### 3.2.1. Teamwork

In the case of teamwork, the results are based on the five related questions:

- Q6_enjoy_woking_with_others: Collaborating with two or more players to achieve a common goal (cooperation and teamwork, co-op missions)?
- Q22_helping_other: Helping other players
- Q28_part_casual_guild: Being part of a friendly, casual guild
- Q25_player_vs._player: Competing with other players
- Q23_getting_know_other: Getting to know other players

**Figure 7:**
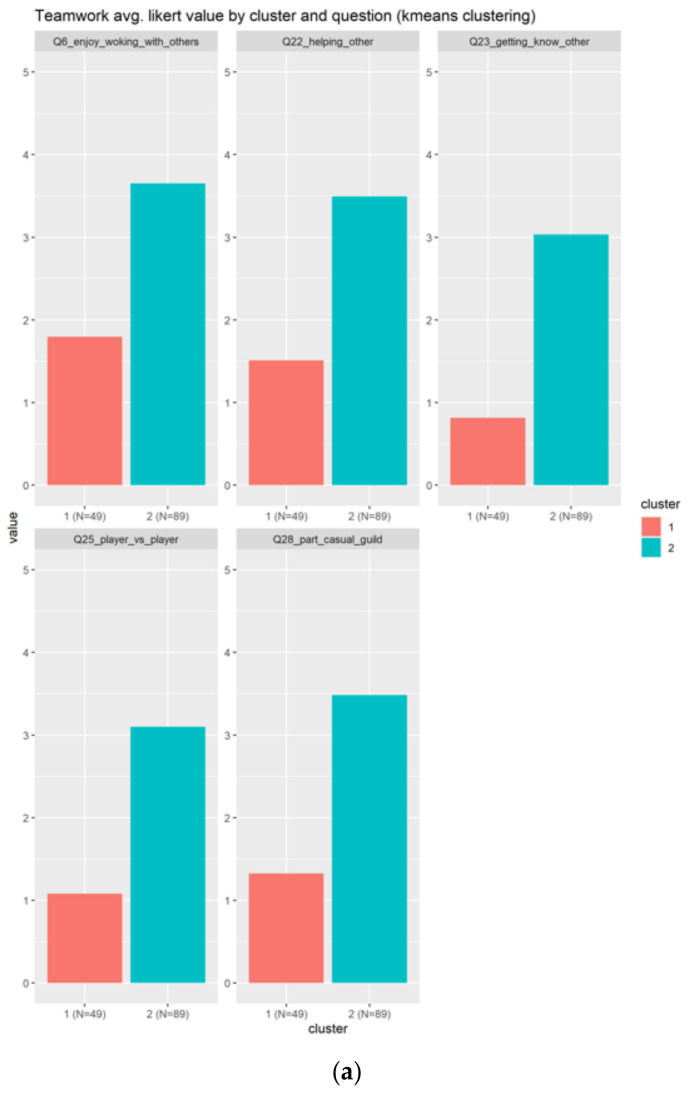

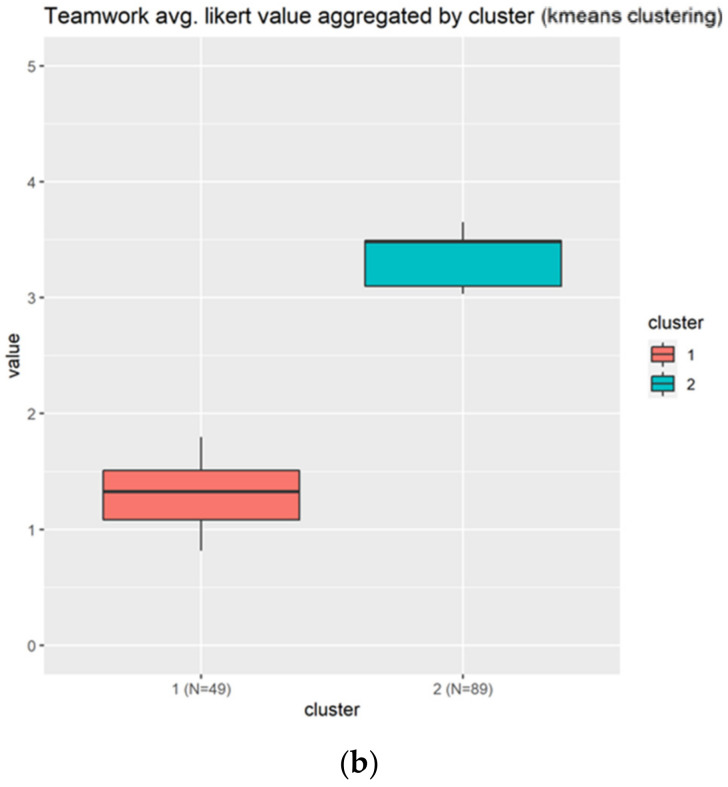
This figure shows the results for the teamwork. Each color identifies a different cluster. The (**a**) subplots shows the results based on each question, meanwhile the (**b**) shows the information condensed in boxplots.

Teamwork generated two clusters, as indicated in Figure 7. Figure 7a, indicates clearly positive feedback (scores bigger than three) across all the questions by the individuals at the cluster two, in blue. The cluster one replies negative to the teamwork elements, because the average score is less than 2. Figure 7b indicates, in terms of boxplots, the results. The blue boxplot of the cluster two indicates that the median score is almost the third quartile (Q3), which indicates that there is less variation in terms of the score from the users that replied almost 4, than the users that replied between 3 and 3.5, which indicate very positive feedback to use teamwork elements. Cluster one has no particular characteristics, rather confirming the disagreement of the individuals inside this group to use teamwork elements. In general, more individuals agreed to use teamwork elements than disagreed (89 vs. 49).

#### 3.2.2. Social

The social element generated more correlation between the questions, which could be because of the thin line that separates the collaboration (teamwork) with the socialization. There are a total of nine related questions:

- Q24_chatting: Chatting with other players
- Q23_getting_know_other: Getting to know other players
- Q32_meaningful_conversations: How often do you find yourself having conversations with other players?
- Q28_part_casual_guild: Being part of a friendly, casual guild
- Q22_helping_other: Helping other players
- Q33_talk_personal_issues: How often do you talk to your online friends about your personal issues?
- Q29_part_serious_guild: Being part of a serious, raid/loot-oriented guild
- Q25_player_vs._player: Competing with other players
- Q39_try_to_provoke_others: How often do you purposefully try to provoke or irritate other players?

**Figure 8:**
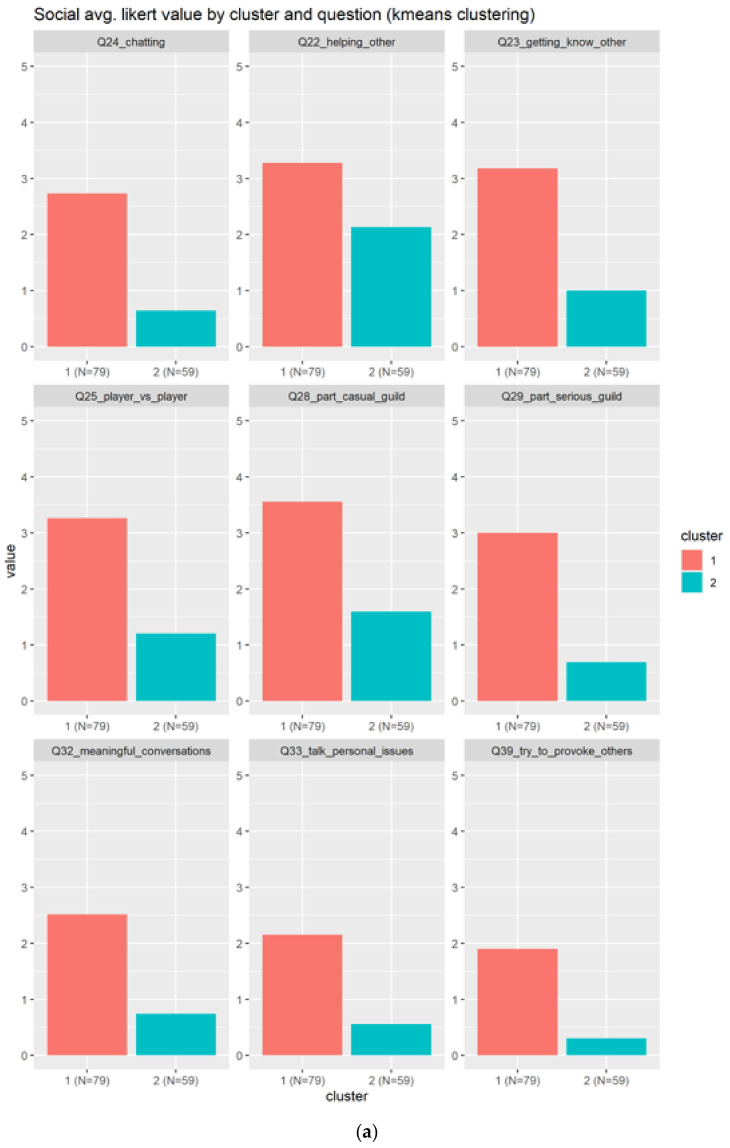

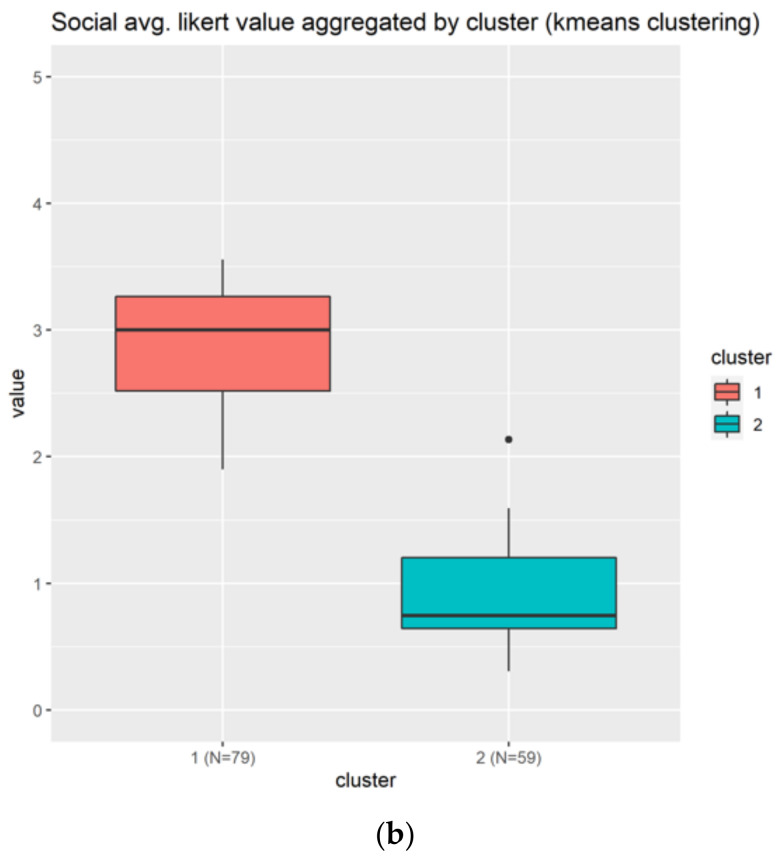
Results for the social related questions. There are two clusters in red and blue. A total of nine questions in subplot (**a**) and two boxplots generated in subplot (**b**).

Figure 8 shows only two clusters, being the bigger one (N = 79) the most positive across the nine questions. As indicated in Figure 8a, the cluster 1 agrees slightly with the use of social elements, meanwhile the cluster 2 in blue totally disagrees (the value is between 0–1). This can indicate either that the social elements are not well represented in our form, or the individuals does not like the social elements, on average.

#### 3.2.3. Rules

In the case of rules, there are only two related questions, which could be because of the design of the form questions that we modified from the original [16] document. The correlated question is reduced to a one, but it is well suited for this case:

- Q18_game_mechanics_rules: Knowing as much about the game mechanics and rules as possible
- Q1_precise_game_mechanics: How interested are you in the precise numbers and percentages underlying the game mechanics?

**Figure 9:**
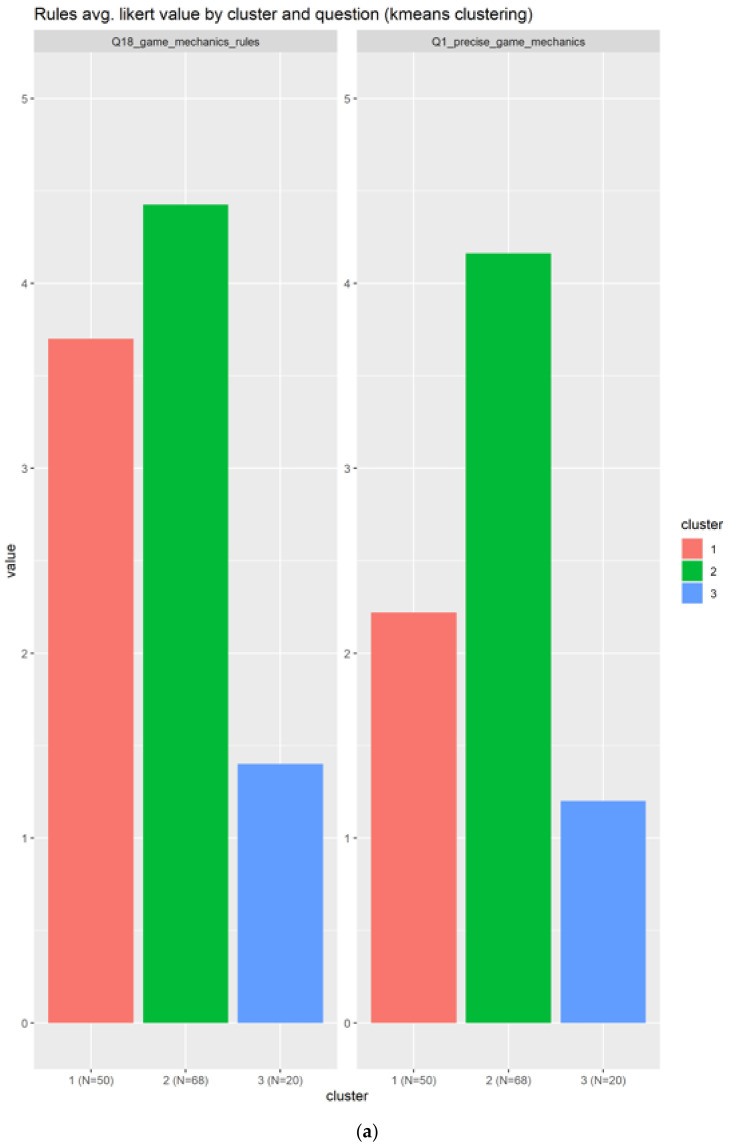

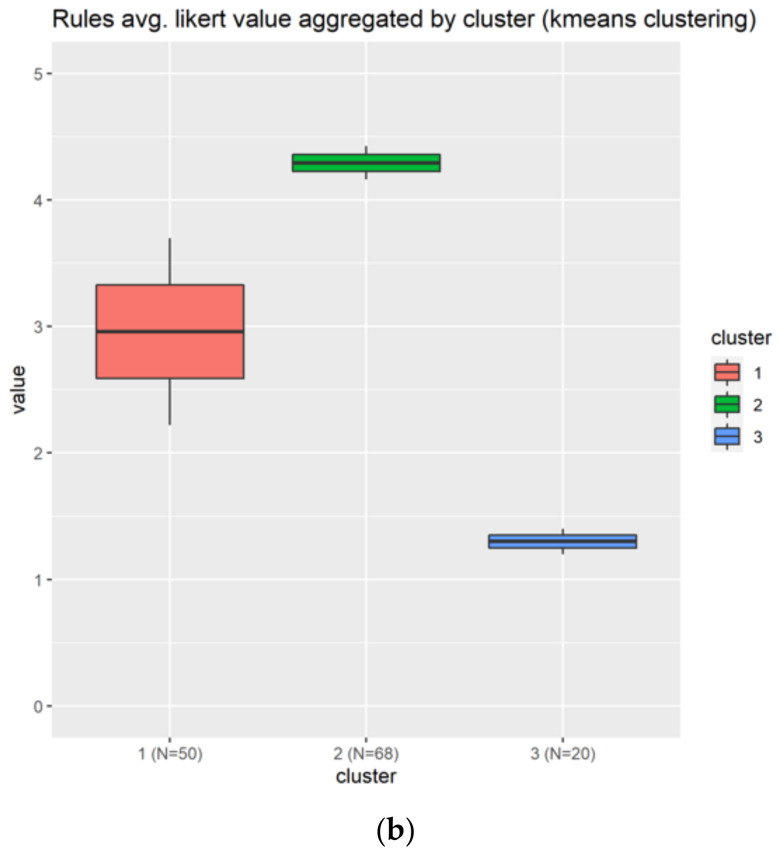
Results for the rules related questions. A total of two related questions in subplot (**a**) and three boxplots generated in subplot (**b**).

In this case (see Figure 9) we have three clusters in red (1), green (2), and blue (3), being the first two well balanced in number of individuals and the last cluster being the smallest one with 20 individuals. Both cluster 1 and 2 are good candidates to represent the rules elements, the second cluster in green being the best candidate with higher scores across the two questions as shown in Figure 9a and also more concentrated boxplot as indicated in Figure 9b.

#### 3.2.4. Progression

Progression has four related questions, and is related to increment your impact inside the game by having more power and/or items to participate into the different missions. The related questions are:

- Q16_becoming_powerful: Becoming powerful
- Q17_accumulate_resources: Accumulating resources and items
- Q14_leveling_character_fast: Leveling up your character as fast as possible
- Q19_self_sufficient_character: Having a self-sufficient character

**Figure 10:**
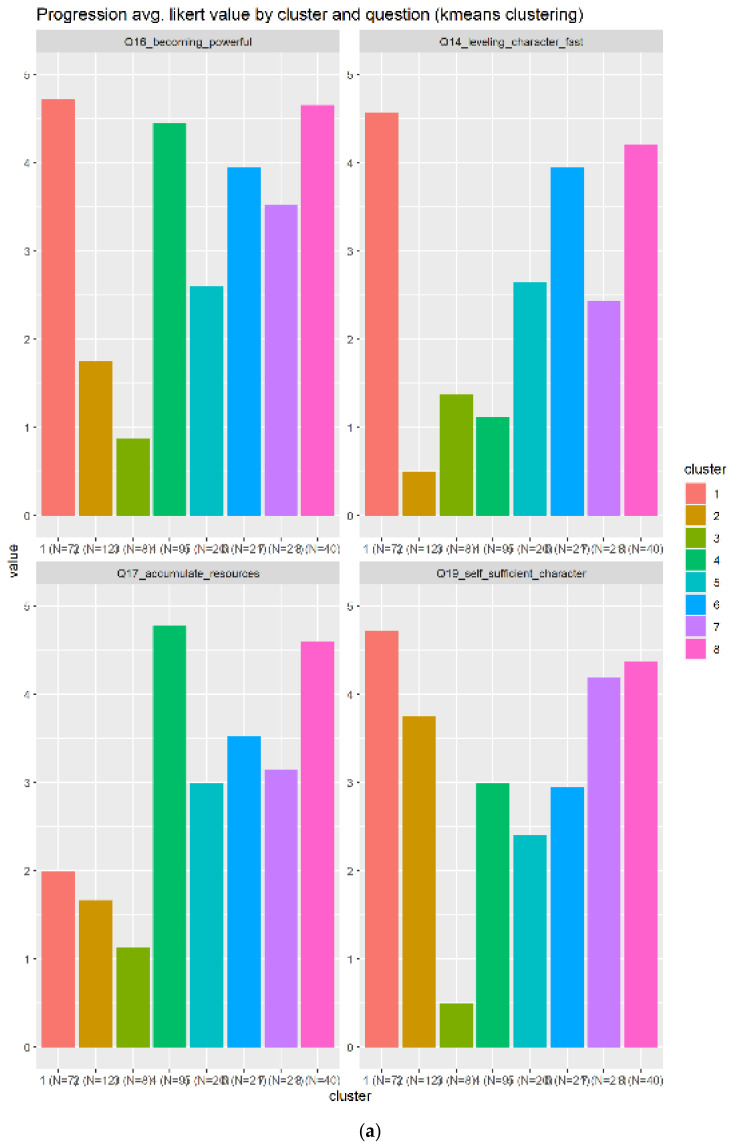

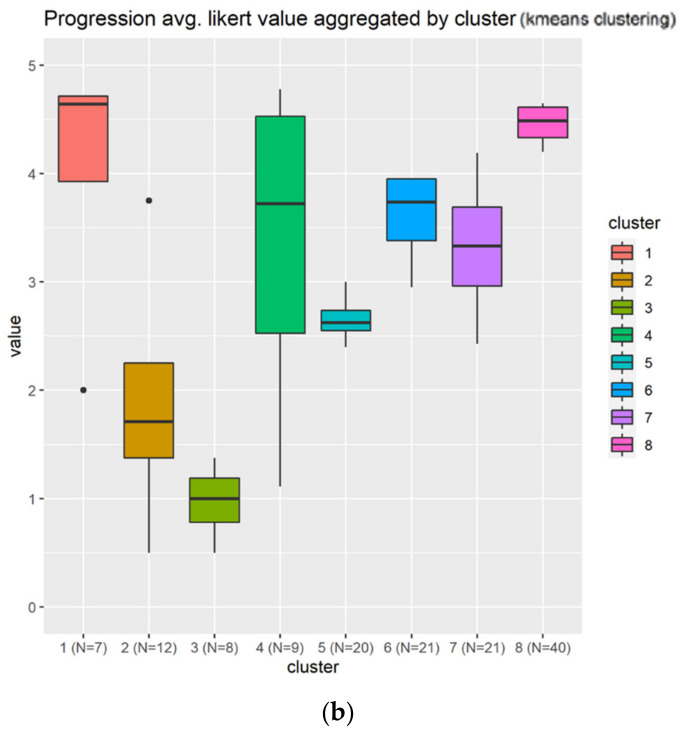
Results for the progression related questions. A total of four related questions in subplot (**a**) and eight boxplots generated in subplot (**b**).

As seen in Figure 10, there are eight clusters with a heterogeneity of opinions about the scores. In this case, the related questions are not as good as we expected to describe the progression elements. We are unable to detect a clear group by simply looking at the Figure 10a, then we have to analyze the Figure 10b to detect the boxplots that are higher in terms of scores (vertical axis) and smaller in terms of IQR. The clusters 1 (red) and 8 (pink) are the candidates to represent the progression elements, the cluster 8 (pink) being the best case because the boxplot IQR is smaller that translates in the same opinion. Additionally, the cluster 8 is bigger (N = 40) than the number 1 (N = 7), including more samples (information).

#### 3.2.5. Points

There are no direct questions related to points elements in our form, instead there are three indirect related questions:

- Q17_accumulate_resources: Accumulating resources and items
- Q16_becoming_powerful: Becoming powerful
- Q15_acquiring_rare_items: Acquiring rare items that most players will never have

**Figure 11:**
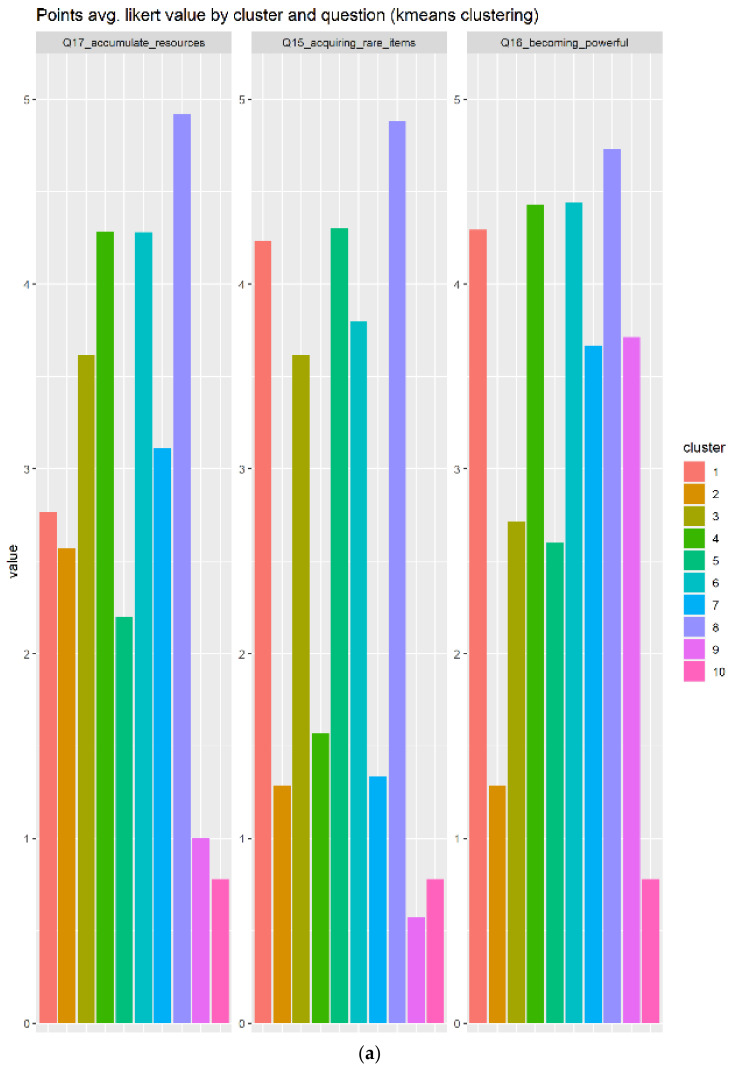

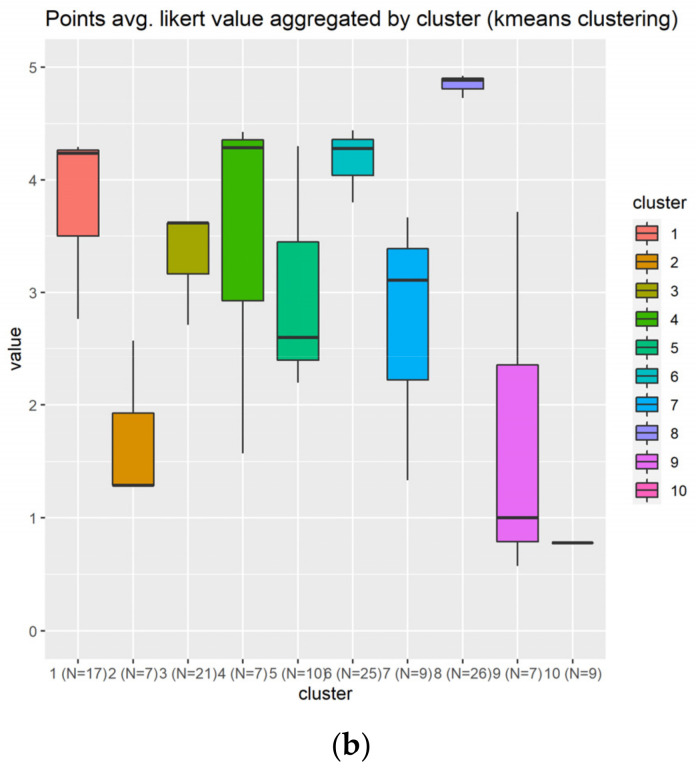
Points results indicate a heterogeneity of opinions; many users replied positively to those questions, as indicated in subplot (**a**). The boxplot (**b**) shows a big IQR in 6 out of 10 cases.

The users’ answers to the points questions, generated a heterogeneity of opinions as show in Figure 11. There are a total of ten clusters, each of them composed more or less with the same portion of users. Cluster 8 in dark violet is distinguished over the others, because the distribution of the answers provided by the users inside are almost the same with the highest value. The boxplot in Figure 11b confirms that behavior. This cluster is composed by N = 26 individuals, which is considerable compared to the other ones.

#### 3.2.6. Narrative

For the case of narrative, there are two questions that are closely related to the term of narrative because it includes immersing the player:

- Q21_escaping_real_world
- Q20_immersed_fantasy_world

**Figure 12:**
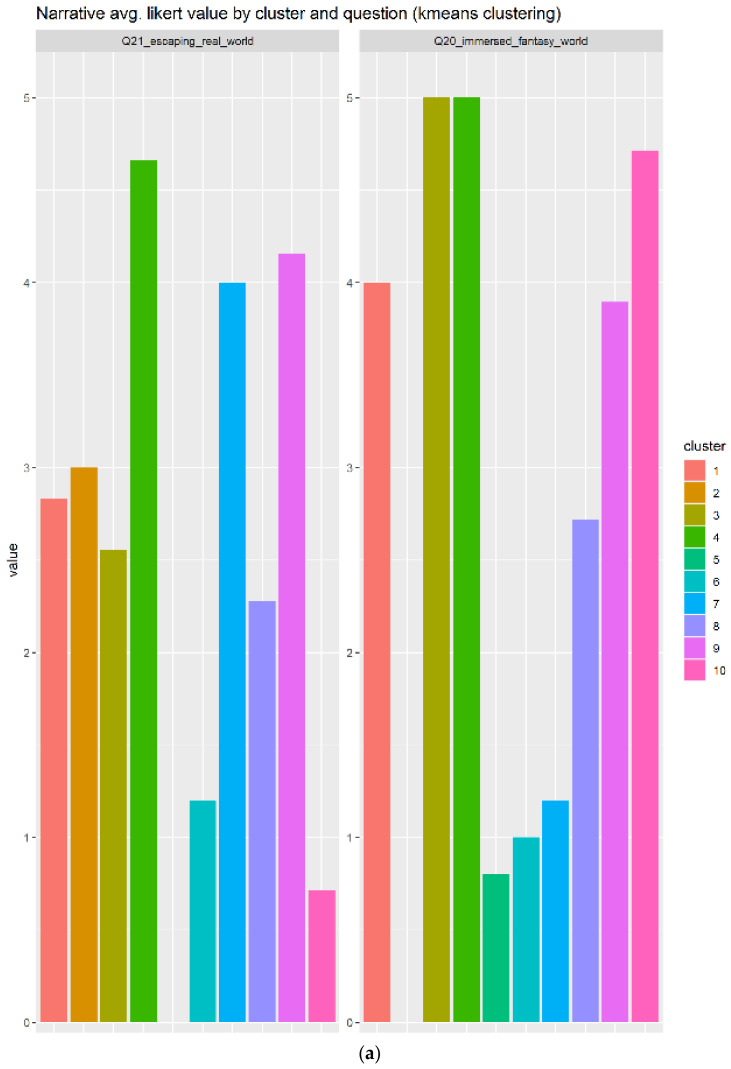

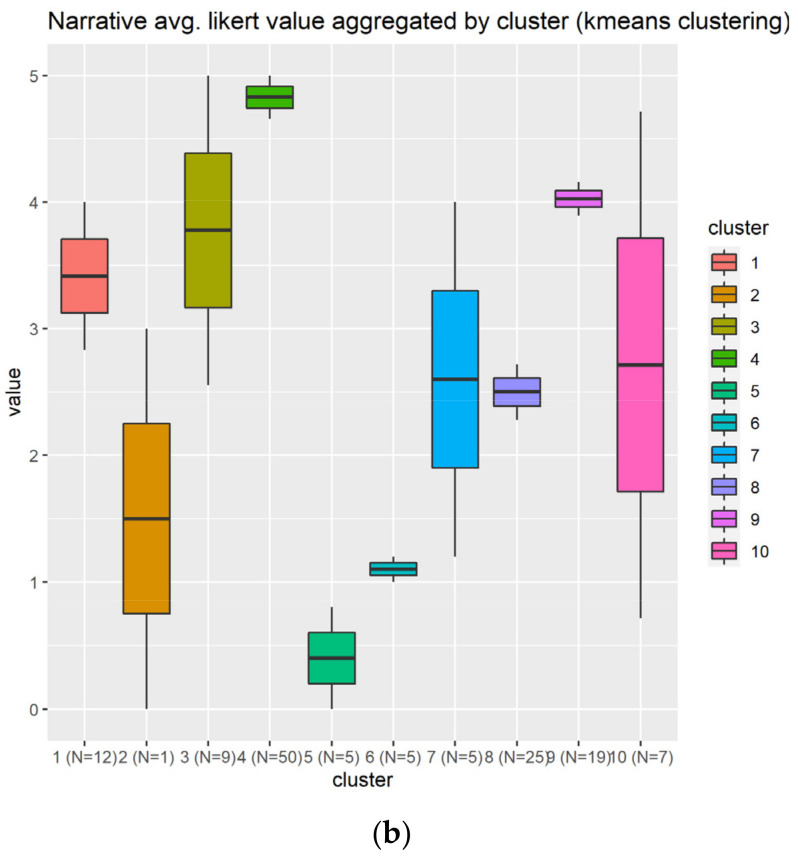
The answer for the narrative element has many clusters (10) indicating a heterogeneity of opinions, as can be seen in subplot (**a**). Boxplots in subfigure (**b**) show 4 of the 10 with a large IQR and 4 of 10 with small IQR, which are the interesting ones.

The narrative also generated a heterogeneity of opinions among the individuals that replied to our form. A total of 10 clusters have been generated, as indicated in Figure 12a, which shows that cluster 4 in green replied very positively to those questions. Additionally, the cluster 9 in light pink replied positively to the narrative elements’ questions. According to the Figure 12b, the best candidate is the cluster 4 in green, because it contains higher scores and a bigger number of individuals (N = 50) compared to the cluster 9 with slightly lower scores in average and smaller number of individuals (N = 19).

#### 3.2.7. Missions

In the case of missions, the related questions are:

- Q6_enjoy_woking_with_others: Collaborating with two or more players to achieve a common goal (cooperation and teamwork, co-op missions)?
- Q22_helping_other: Helping other players
- Q28_part_casual_guild: Being part of a friendly, casual guild
- Q25_player_vs._player: Competing with other players
- Q23_getting_know_other: Getting to know other players

**Figure 13:**
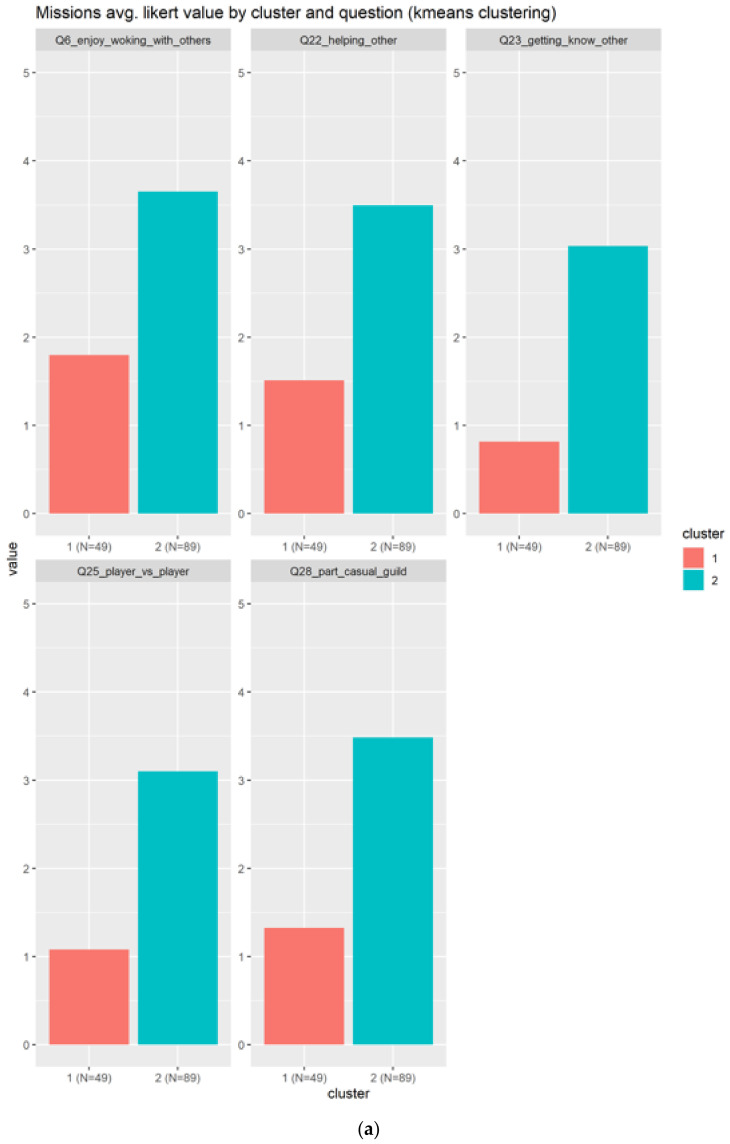

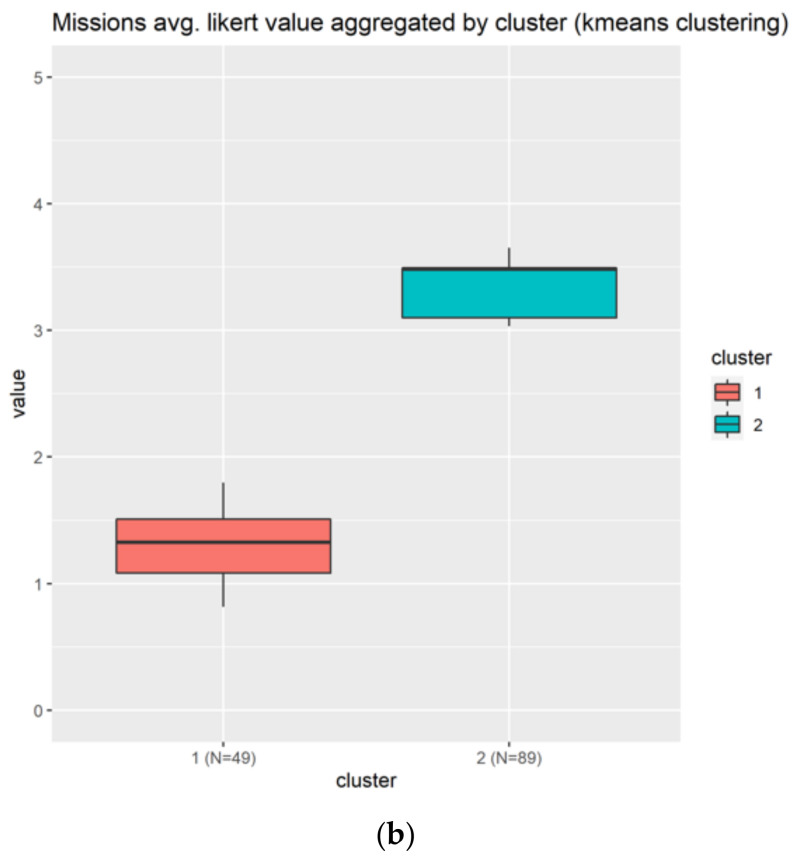
Results for the mission elements. Only two clusters are generated. The (**a**) subplots shows the results based on each question, meanwhile the (**b**) shows the information condensed in boxplots.

In the case of the missions, the questions are more related to teamwork rather than missions, but there is a small component to take into consideration, which is a common goal. Here the results are less relevant, because the average score is 3 as indicated in Figure 13a, which slightly agrees with the mission related questions. Figure 13b confirms that behavior, because the important cluster indicated at the blue boxplot is closer to a score of three.

#### 3.2.8. Level

Level has two related questions. The first one, the key element, has a clear relation to leveling elements. The second element is a side-effect of leveling the character in the games:

- Q14_leveling_character_fast: Leveling up your character as fast as possible
- Q16_becoming_powerful: Becoming powerful

**Figure 14:**
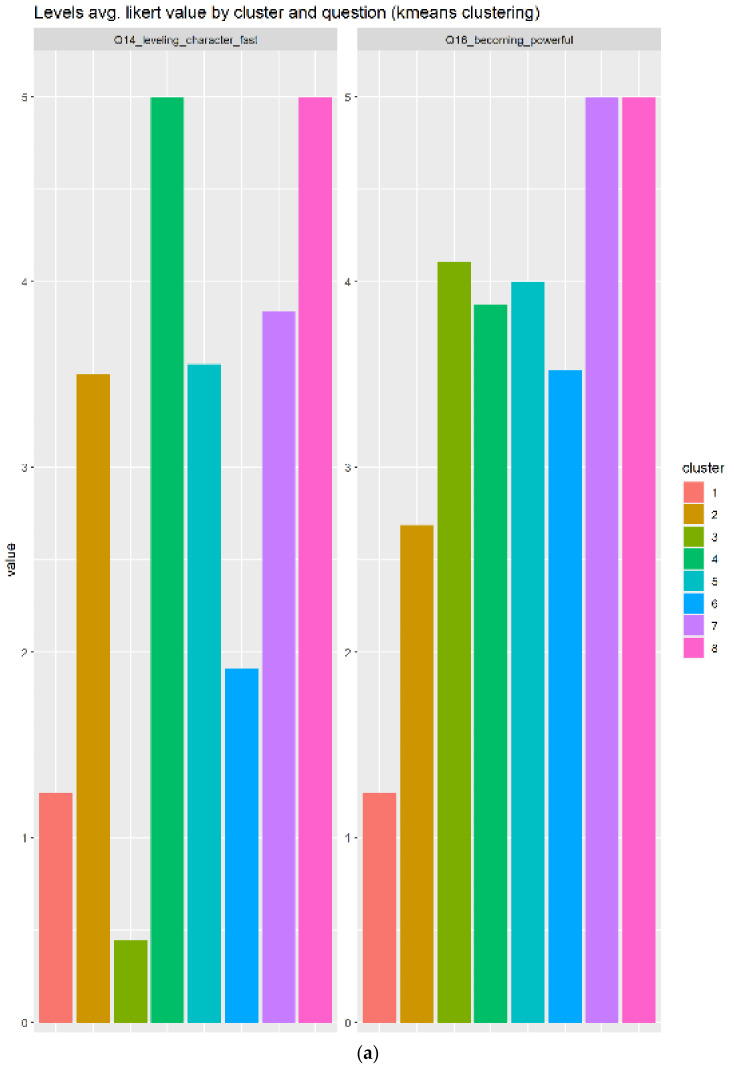

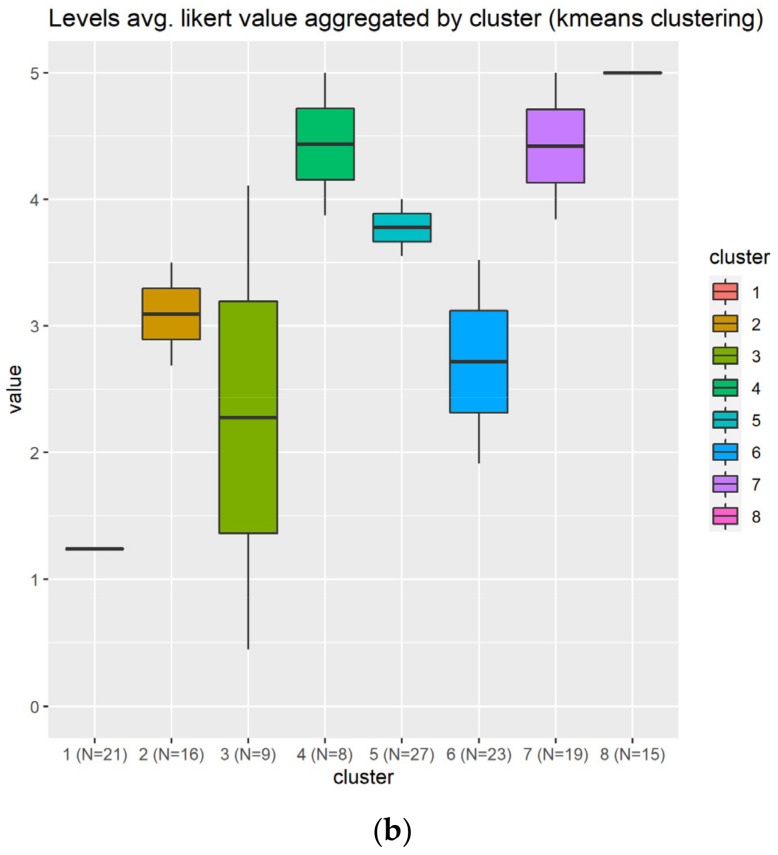
Results for leveling elements. Eight cluster generated, as can be seen in subfigure (**a**), where three of them are important according to the boxplots in subfigure (**b**).

Figure 14 indicates a total of eight clusters, where three of those responded positively to the leveling elements, meanwhile the other elements remain neutral (almost 3 in average), except one (cluster 1) which responded negatively to the leveling elements. Figure 14b shows three clusters as the best candidates (4, 7, and 8), but the cluster eighth is the most optimal, because it has the smallest IQR and the highest average score.

#### 3.2.9. Goals

In the case of goals, the results based on the three related questions:

- Q13_collecting_distinctive_objects: How much do you enjoy collecting distinctive objects or clothing that have no functional value in the game?
- Q15_acquiring_rare_items: Acquiring rare items that most players will never have

**Figure 15:**
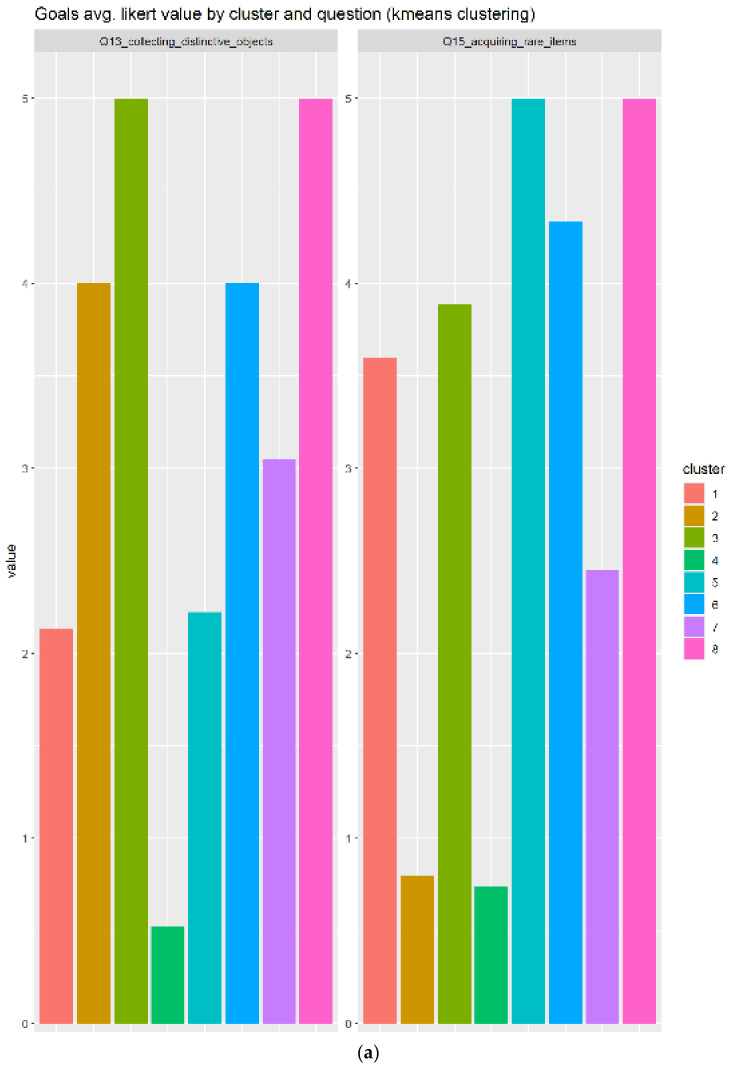

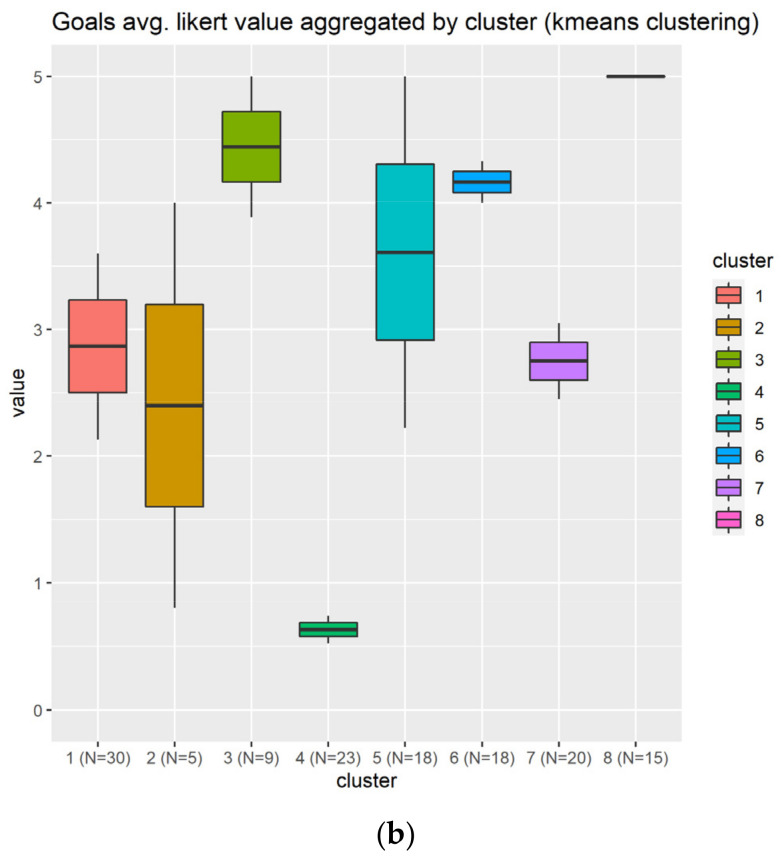
The goals generated eight cluster groups, where only three of them respond positively to those elements. Subfigure (**a**) indicate the two questions in detail, meanwhile (**b**) shows the boxplots of the groups.

This time, eight clusters have been generated according to Figure 15a, which reveals that cluster 8 is the most appropriated group to associate with goals elements, as this contains the individuals that responded almost the highest possible score (5/5). The boxplot in Figure 15b confirms that, with one of the smallest IQR presented on the study. This cluster contains enough individuals (N = 15) to be considered as representative. 

#### 3.2.10. Badges

In the case of badges, the results are based on the three related questions:

- Q13_collecting_distinctive_objects: How much do you enjoy collecting distinctive objects or clothing that have no functional value in the game?
- Q15_acquiring_rare_items: Acquiring rare items that most players will never have
- Q17_accumulate_resources: Accumulating resources and items

**Figure 16:**
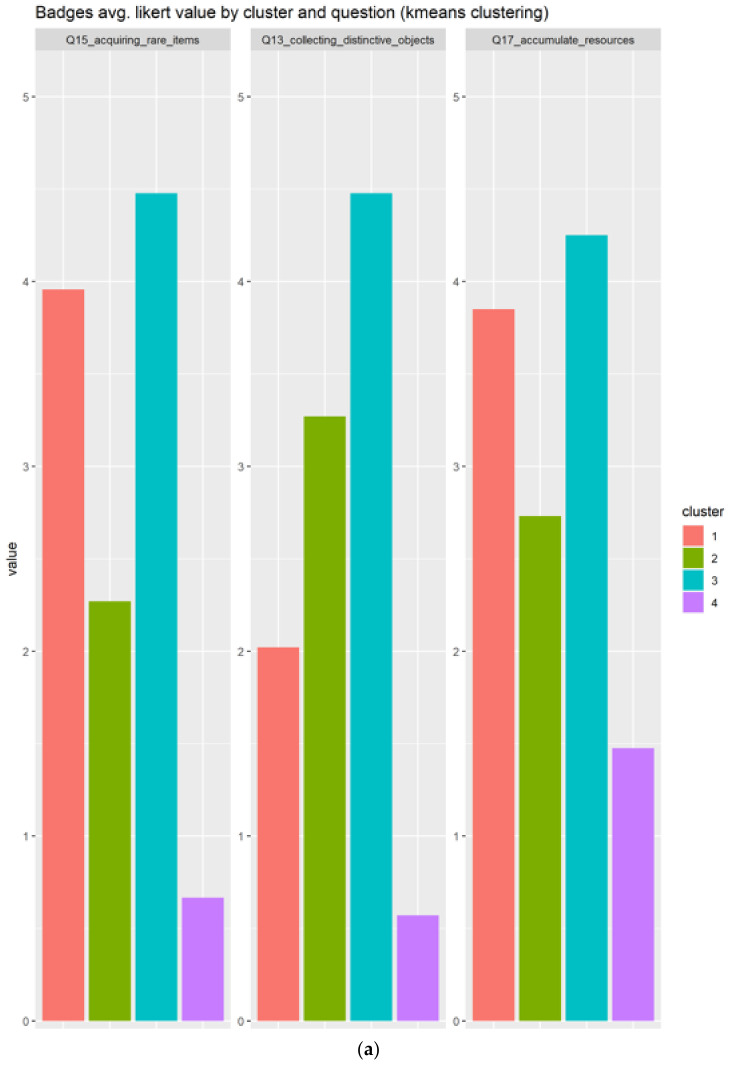

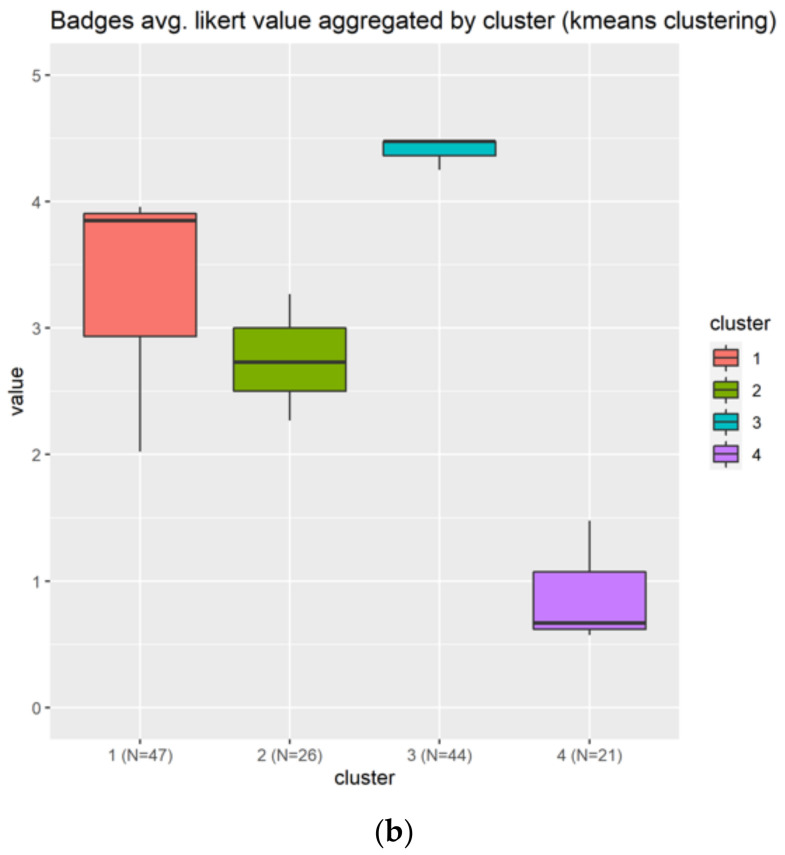
This figure shows the results for the Badges. Each color identifies a different cluster. The (**a**) subplots show the results based on each question, meanwhile the (**b**) shows the information condensed in boxplots.

As depicted in Figure 16a, we can see that cluster 1 and 3 responds very positively to acquiring rare items and accumulate resources, but only cluster 3 very positively evaluates the collection of distinctive objects. Next, we can determine that the cluster 3 will be the best candidate for users with a profile compatible with badges.

Analyzing the data in a summarized way in the boxplots in Figure 16b, we can confirm that the cluster 3 in cyan color, made up of 44 individuals, clearly represents this element, since the responses are quite positive (4.5/5), and the IQR of the Boxplot has a small value, so many individuals coincide.

#### 3.2.11. Competition

In the case of competition there are seven selected questions, variables, detailed as follows:

- Q25_player_vs._player: Competing with other players
- Q26_dominate_kill_others: Dominating/killing other players
- Q23_getting_know_other: Getting to know other players
- Q29_part_serious_guild: Being part of a serious, raid/loot-oriented guild
- Q24_chatting: Chatting with other players
- Q6_enjoy_woking_with_others: Collaborating with two or more players to achieve a common goal (cooperation and teamwork, co-op missions)?
- Q31_annoying_others: Doing things that annoy other players

**Figure 17:**
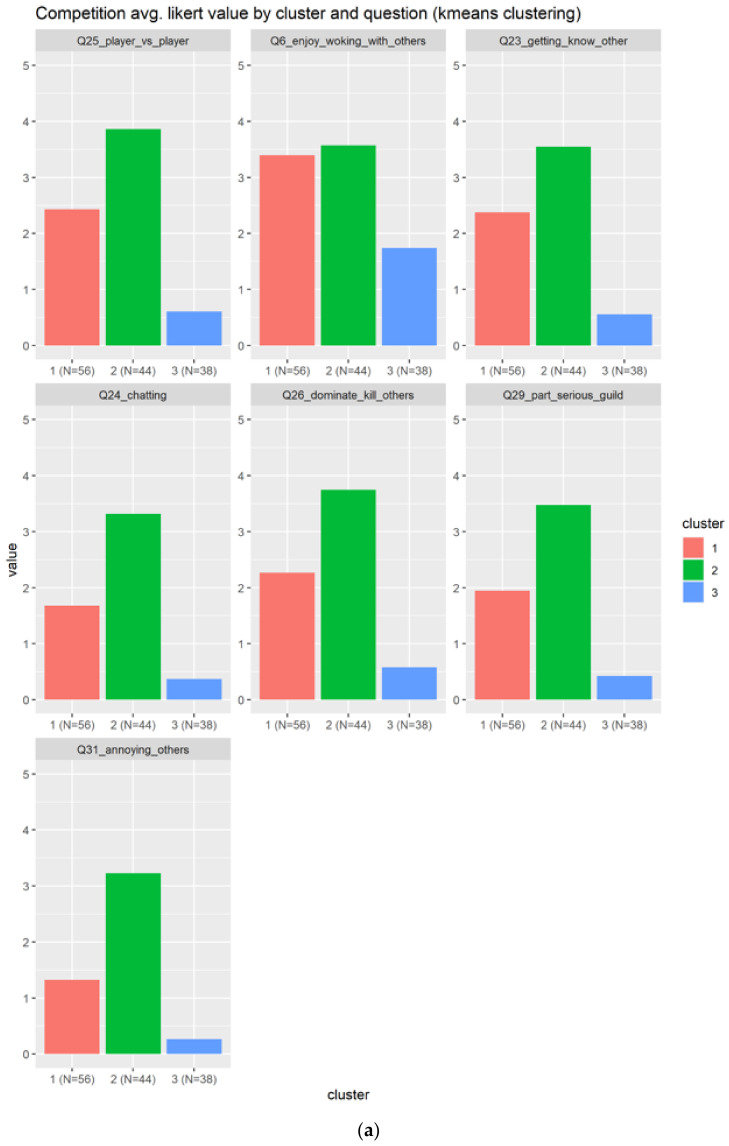

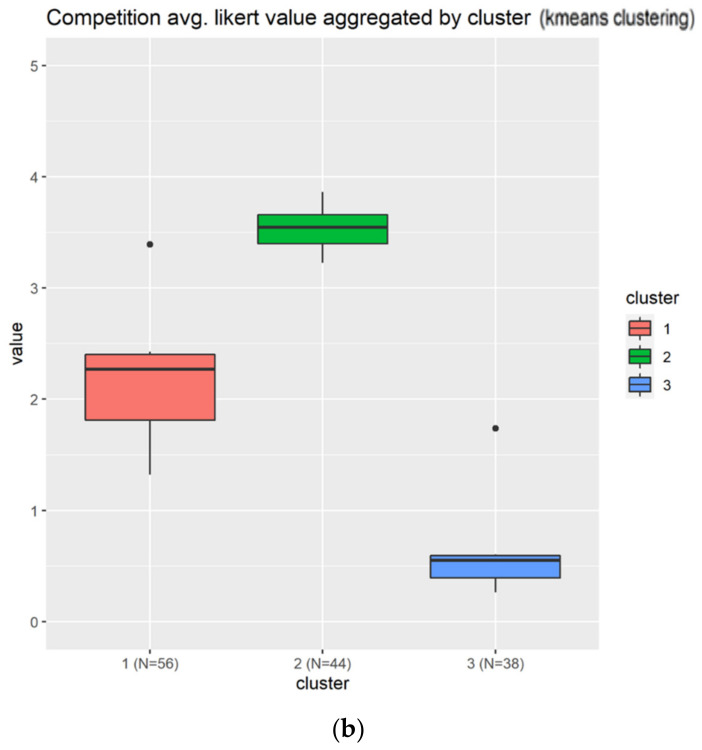
This figure shows the results for the Competition. There are three clusters in different colors. The (**a**) shows the results based on each question, meanwhile the (**b**) shows the information condensed in boxplots.

We can observe in Figure 17a that there are a total of three groups, having the second cluster as the most positive one, in green color, because its average value is bigger than three across all the questions. The cluster three is composed of the individuals that do not like the competition elements. Figure 17b confirms the information in the condensed boxplots, the boxplot in green that represents the second cluster is higher than the others, meanwhile the blue one is almost to the zero level. The cluster two will be choose as the best representative for the competition gamification element.

#### 3.2.12. Customization

For the customizations there are only three related questions, as indicated in the following:

- Q8_time_customizing_character: How much time do you spend customizing your character during character creation?
- Q3_use_character_builder: How often do you use a character builder? (0 never, 5 always)
- Q9_character_outfit_matches_style: How important is it to you that your character’s outfit matches in color and style?

**Figure 18:**
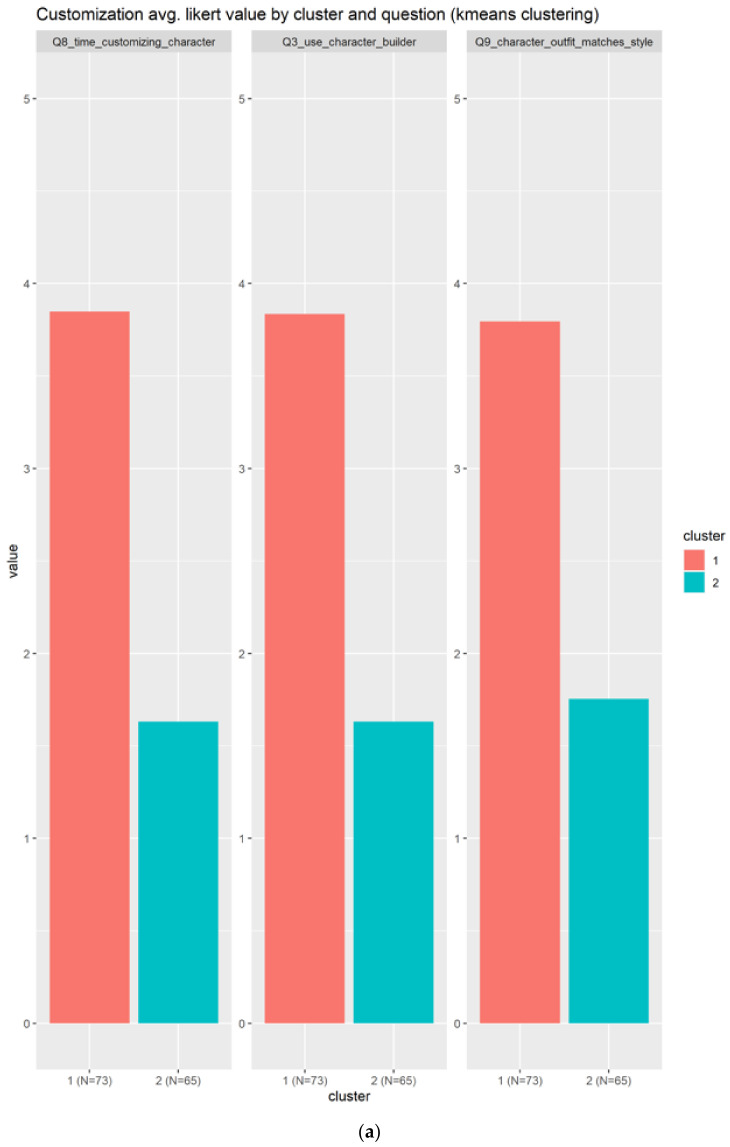

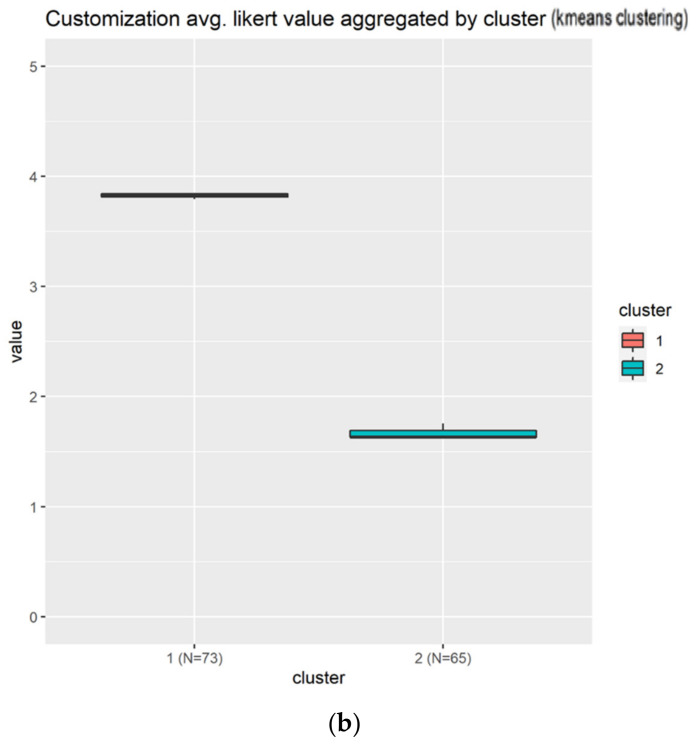
Results from customization. There are only two clusters, with a clear separation. The (**a**) subplots show the results based on each question, meanwhile the (**b**) shows the information condensed in boxplots.

Customization is composed by only two clusters as indicated in Figure 18. Those clusters are almost the same size in terms of individuals, something that is good, because the groups are well balanced. The first group in red, cluster 1, is composed of those individuals that agree with the customization elements, as the average value in all the questions is almost 4/5, as can be seen in Figure 18a. The other cluster in blue is composed of those who slightly disagree with the customization’s elements. Figure 18b confirms that the cluster 1 agrees with a unanimous opinion about the use of customization elements.

### 3.3. Determining the Game Genre Related to Each Gamification Element

**Figure 19:**
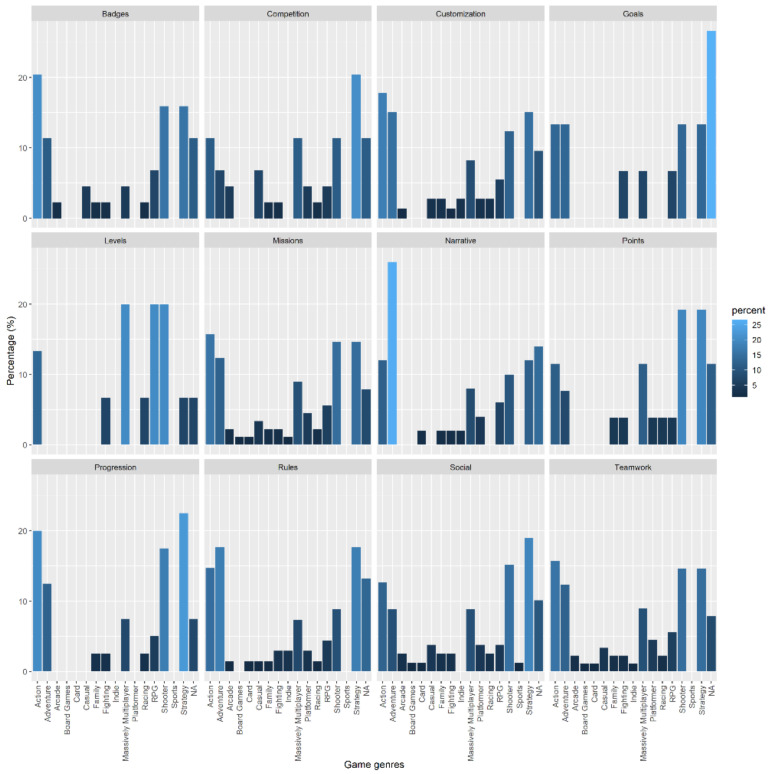
Bar chart showing the percentage of appearance of each game genre in each type of gamification element, a lighter color indicates a greater number of appearances.

**Figure 20:**
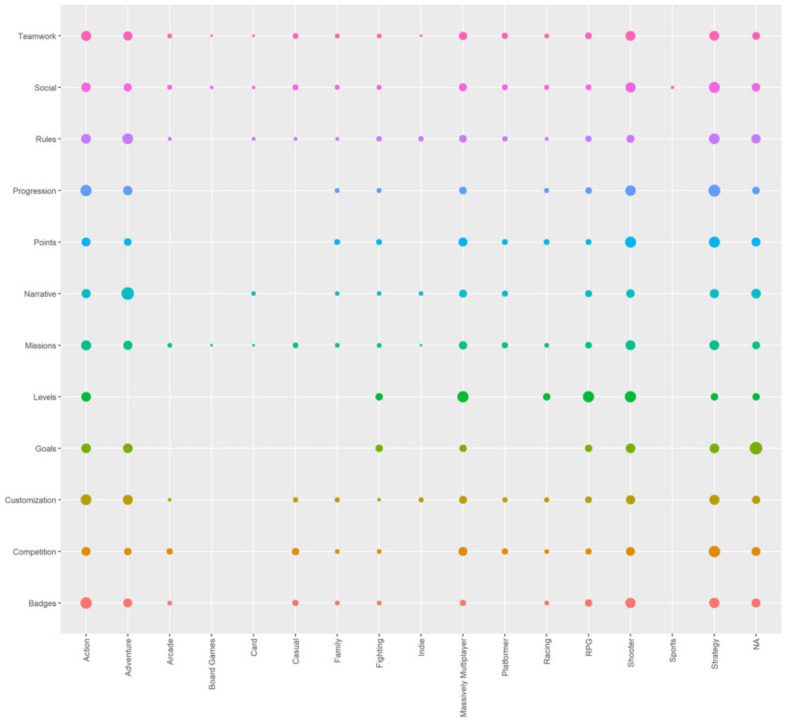
Points graph showing the percentage of appearance of each game genre in each type of gamification element by the size of the point, the color indicates the game genre.

In this subsection we have condensed the information related to the preferred game genre for each specific cluster of each gamification element. Figure 19 and Figure 20 show the results in the most summarized way possible. As indicated in the methodology, to generate those results we have selected one cluster for each gamification element based on the results of the previous Section 3.

Figure 19 shows the bars for each of the elements in bar graphs separated into 3 × 4 columns. The intensity is shown in a blue scale; a lighter blue indicates that the users who play the game genre indicated on the vertical axis are more in agreement. Figure 20 concentrates the same information as Figure 5, but in a way that is easier to interpret. The bigger each point is, the more important it is. The color of each point indicates a different gamification element (one color for each row).

## 4. Discussion

Before conducting the study, we have surveyed the literature for tools to help us build the form with the different questions. After a wide search, we have found the study provided by [16]. We have decided to recompile the questions provided in this study. evaluated the possibility of generating new indicators based on the game genre that the player usually plays. For this, we must check if there are groups of users and how they are polarized based on the previous questions, examining if it was possible to detect differences in behavior. We have found that determining the key or reference questions is a critical step in the study, since it involves a human factor that interprets and decides the question that best represents the gamification element in question.

After this, finding related questions, where users respond similarly, is relatively easy and straightforward through simple correlation. This process has produced cases with two cases and other cases with multiple related questions. We expected that the greater the number of related questions, the fewer groupings we would obtain as more information was available to the user. According to the results, there is no relationship between the number of questions available for each element and the number of users grouped.

We can see how on some gamification elements, there is a generation of two or three groups where the users are polarized between a positive score, reflecting that they agree, and a negative or neutral one where disagreement or neutrality is shown. We have these cases in 6 out of 12, exactly in half of, the cases. However, we get more than 4 groups in the remaining elements, showing a diversity of opinions. Therefore, we could conclude that in those 6 cases, teamwork, social, rules, missions, competition, and personalization are elements that can be clearly described with the questions used in our study. We could also conclude that the users on whom the questionnaire has been made are easily polarized based on these elements. As far as those elements are concerned, it can keep a part of the users and make the other portion decide to stop using the gamified system with those elements.

Analyzing the results of Figure 20, reading the results in rows (horizontally), we can analyze for each one of those cases the most relevant game genre. The most relevant game genre is determined by the size of the dot in each intersection between the horizontal term (Gamification element) and vertical term (Game genre). For instance, we can see that the three biggest dots in the case of Teamwork are intersecting vertically with Action, Shooter, Strategy, and Adventure. A more detailed view of this can be seen in Figure 19, where the highest bars for Teamwork subplot are those indicated above, having at least 10% of the relative percentage assigned to each one.

In the case of Social, we can see a general picture in the Figure 20, where the biggest dots are related to Action, Strategy and Shooter, one item less than the previous one. That we can confirm in detail, in Figure 19, where all of those fulfills having a relative percentage bigger than 10%. Following this approach, we have determined the following association between gamification element and the kind of game genre played by the user, the order of the game genres matters, >means that the left element is more important than the right, = means that the left element and the right have the same importance. The element indicated with *, indicates a relative percentage bigger than 15%, which means it is the possible main genre for that gamification element:

- Teamwork: *action > shooter = strategy > adventure
- Social: *strategy > *shooter > action
- Rules: *adventure > *strategy > action
- Missions: *action > strategy = shooter > adventure
- Competition: *strategy >action = massive multiplayer = shooter
- Customization: *action > adventure = strategy > shooter

With the accumulated knowledge about the genres and the gamification elements, we can proceed straightforwardly with just one question about the game that the users play to decide which gamification element to get positive feedback on the gamification system. Another detail that we have noticed in the study is that we observe no clear relationship between a gamification element related to a game genre. In other words, there is not an element that is valued equally by the different people who play each of the genres, neither in the affirmative case (larger circle) nor in the negative case (small or missing circle). However, we observe that for some game genres such as action, strategy, shooter, adventure, and MMO, all the gamification elements are represented to a greater extent than other genres, so it is easier to implement gamification in users that play any of these genres.

## 5. Conclusions

We have been able to capture the different profiles of the users who have responded to the form. These profiles have been represented in different clusters of different sizes and numbers for each gamification element. In some cases, we obtain a cluster of uses who clearly agree with the questions representing a specific gamification element. In other cases, we obtain responses from profiles that do not agree with the use of mission elements or a slight indifference (half of the Likert scale). On the other hand, we have been able to identify the preferred types of games according to the gamification element. In the near future, it would be necessary to know how recurrent these players are in the genres that indicate how much they play.

## Figures and Tables

**Table 1:** Relation of gamification elements (GE).

GE	Description
Teamwork	Combined action of a group of players, especially when efficient and effective.
Social	Related to the interaction with other players, especially for pleasure.
Rules	Statements that tell players what is or is not allowed in a particular situation.
Progression	Allows players to locate themselves (and their progress) within a game. Examples: progress bars, maps, steps.
Points	Unit used to measure player performance. Examples: scores, number of kills, experience points.
Narrative	Order of events happening in a game, i.e., choices influenced by player actions. Examples: strategies the player uses to go through a level (stealth or action), which also influence the ending.
Missions	An important assignment given to a player or group of players, typically involving doing something.
Levels	Hierarchical game layers, providing a gradual way for players to obtain new advantages upon advancing. Examples: character levels, skill level.
Goals	The object of a person’s ambition or effort; an aim or desired result.
Customization	The action of modifying something to suit a particular individual or task.
Competition	When two or more players compete against each other to achieve a certain common goal. Examples: Player vs. Player, scoreboards, conflict.
Badges	Symbolic rewards given to players for their achievements, such as acing a skill. Badges help players feel recognized for their efforts.

**Table 2:** Relation of gamification element with the key question and questions with a correlation factor greater than 50%.

Gamif. Element	Key Question	Related Question	Correlation
Teamwork	Q6_enjoy_woking_with_others	Q22_helping_other	0.575
		Q28_part_casual_guild	0.524
		Q25_player_vs._player	0.518
		Q23_getting_know_other	0.516
Social	Q24_chatting	Q23_getting_know_other	0.851
		Q32_meaningful_conversations	0.671
		Q28_part_casual_guild	0.66
		Q22_helping_other	0.549
		Q33_talk_personal_issues	0.545
		Q29_part_serious_guild	0.542
		Q25_player_vs._player	0.533
		Q39_try_to_provoke_others	0.524
Rules	Q18_game_mechanics_rules	Q1_precise_game_mechanics	0.541
Progression	Q16_becoming_powerful	Q17_accumulate_resources	0.575
		Q14_leveling_character_fast	0.535
		Q19_self_sufficient_character	0.502
Points	Q17_accumulate_resources	Q16_becoming_powerful	0.575
		Q15_acquiring_rare_items	0.575
Narrative	Q21_escaping_real_world	Q20_immersed_fantasy_world	0.544
Missions	Q6_enjoy_woking_with_others	Q22_helping_other	0.575
		Q28_part_casual_guild	0.524
		Q25_player_vs._player	0.518
		Q23_getting_know_other	0.516
Levels	Q14_leveling_character_fast	Q16_becoming_powerful	0.535
Goals	Q13_collecting_distinctive_objects	Q15_acquiring_rare_items	0.554
Customization	Q8_time_customizing_character	Q3_use_character_builder	0.588
		Q9_character_outfit_matches_style	0.513
Competition	Q25_player_vs._player	Q26_dominate_kill_others	0.811
		Q23_getting_know_other	0.617
		Q29_part_serious_guild	0.614
		Q24_chatting	0.533
		Q6_enjoy_woking_with_others	0.518
		Q31_annoying_others	0.516
Badges	Q15_acquiring_rare_items	Q17_accumulate_resources	0.575
		Q13_collecting_distinctive_objects	0.554

**Table 3:** Relation of the complete question title to the short term used in the study.

Short Title	Title
Q1_precise_game_mechanics	How interested are you in the precise numbers and percentages underlying the game mechanics?
Q2_character_optimized	How important is it to you that your character is as optimized as possible for their profession/role?
Q3_use_character_builder	How often do you use a character builder? (0 never, 5 always)
Q4_grouped_soloing	Would you rather be grouped (0) or soloing (5)?
Q5_character_solo_well	How important is it to you that your character can solo well?
Q6_enjoy_woking_with_others	Collaborating with two or more players to achieve a common goal (cooperation and teamwork, co-op missions)?
Q7_wellknown_in_game	How important is it to you to be well-known in the game?
Q8_time_customizing_character	How much time do you spend customizing your character during character creation?
Q9_character_outfit_matches_style	How important is it to you that your character’s outfit matches in color and style?
Q10_character_looks_different	How important is it to you that your character looks different from other characters?
Q11_explore_to_complete_the_world	How much do you enjoy exploring the world just for the sake of exploring it?
Q12_finding_uncommon_quests_locations	How much do you enjoy finding quests, or locations that most people do not know about?
Q13_collecting_distinctive_objects	How much do you enjoy collecting distinctive objects or clothing that have no functional value in the game?
Q14_leveling_character_fast	Leveling up your character as fast as possible
Q15_acquiring_rare_items	Acquiring rare items that most players will never have
Q16_becoming_powerful	Becoming powerful
Q17_accumulate_resources	Accumulating resources and items
Q18_game_mechanics_rules	Knowing as much about the game mechanics and rules as possible
Q19_self_sufficient_character	Having a self-sufficient character
Q20_immersed_fantasy_world	Being immersed in a fantasy world
Q21_escaping_real_world	Escaping from the real world
Q22_helping_other	Helping other players
Q23_getting_know_other	Getting to know other players
Q24_chatting	Chatting with other players
Q25_player_vs._player	Competing with other players
Q26_dominate_kill_others	Dominating/killing other players
Q27_explore_map_zone_world	Exploring every map or zone in the world
Q28_part_casual_guild	Being part of a friendly, casual guild
Q29_part_serious_guild	Being part of a serious, raid/loot oriented guild
Q30_trying_new_roles	Trying out new roles and personalities with your characters
Q31_annoying_others	Doing things that annoy other players
Q32_meaningful_conversations	How often do you find yourself having conversations with other players?
Q33_talk_personal_issues	How often do you talk to your online friends about your personal issues?
Q34_receive_support_reallife_problems	How often have your online friends offered you support when you had a real life problem?
Q35_make_stories_for_your_character	How often do you make up stories and histories for your characters?
Q36_role_play_characters	How often do you role-play your character?
Q37_play_to_avoid_reallife_problems	How often do you play so you can avoid thinking about some of your real-life problems or worries?
Q38_play_to_relax	How often do you play to relax from the day’s work?
Q39_try_to_provoke_others	How often do you purposefully try to provoke or irritate other players?
U1_economy_accumulate_money	Accumulating money
U2_economy_transactions_exchanges_within_the_game	Acquiring transactions or exchange within the game
U3_imposed_choice_advance_in_the_game	Do you like take obliged decisions in order to advance in the game?
U4_imposed_choice_judgements_different_narrative	How important is it to you take judgements, forced choices (different from Narrative) in the game.
U5_time_pressure_countdowns_timer	How important is it to you have pressure through time in-game (countdowns, timer)?
U6_social_pressure	You enjoy peer pressure through social interactions with another players?

## Data Availability

The data that support the findings of this study are available from the corresponding author, (R.S.C.-E.), upon reasonable request.
